# Myelin oligodendrocyte glycoprotein antibody-associated optic neuritis: an update

**DOI:** 10.5935/0004-2749.20230012

**Published:** 2023

**Authors:** Katharina Messias, Vanessa Daccach Marques, Andre Messias

**Affiliations:** 1 Department of Neurosciences and Behavioral Sciences, Faculdade de Medicina de Ribeirão Preto, Universidade de São Paulo, Ribeirão Preto, SP, Brazil.; 2 Department of Ophthalmology, Otorhinolaryngology and Head and Neck Surgery, Faculdade de Medicina de Ribeirão Preto, Universidade de São Paulo, Ribeirão Preto, SP, Brazil.

**Keywords:** Myelin oligodendrocyte glycoprotein, Multiple sclerosis, Neuromyelitis optica, Optic neuritis, Glicoproteína mielina-oligodendrócito, Esclerose múltipla, Neuromielite óptica, Neurite óptica

## Abstract

Myelin oligodendrocyte glycoprotein-immunoglobulin G (IgG)-associated optic neuritis has been established as a new entity of immune-mediated optic neuropathy. Patients usually present with recurrent optic neuritis, often bilaterally with initially severe vision loss and optic disc edema. However, in contrast to aquaporin 4-IgG-seropositive neuromyelitis optica spectrum disorder, visual recovery tends to be more favorable, with good response to steroid treatment. Another important differential diagnosis of myelin oligodendrocyte glycoprotein-IgG--associated optic neuritis is multiple sclerosis. Close monitoring for signs of relapse and long-term immunosuppression may be considered to maintain optimal visual function. The diagnosis can be made on the basis of the presence of a specific, usually serological, antibody against myelin oligodendrocyte glycoprotein (IgG; cell-based assay), and a demyelinating event (optic neuritis, myelitis, brainstem syndrome, or cortical lesions with seizures). The clinical spectrum of this newly recognized inflammatory demyelinating disease is expanding rapidly. We briefly review the epidemiological characteristics, clinical manifestations, diagnostic considerations, and treatment options of myelin oligodendrocyte glycoprotein-IgG-associated optic neuritis.

## Abstract (pt)

A neurite óptica associada à glicoproteína de oligodendrócito de mielina-IgG foi estabelecida como uma nova entidade de neuropatia óptica imunomediada. Tipicamente os pacientes apresentam neurite óptica recorrente, muitas vezes bilateral, com perda de visão frequentemente severa e alta prevalência de edema do disco óptico na fase aguda. No entanto, em contraste com *neuromyelitis optica spectrum disorder* associada com presença de anticorpo contra aquaporina 4, a recuperação visual tende a ser mais favorável e responde bem ao tratamento com corticoide em altas doses. A esclerose múltipla representa outro importante diagnóstico diferencial de glicoproteína de oligodendrócito de mielina-IgG. O diagnóstico pode ser feito com base na presença de um anticorpo específico, geralmente sorológico contra glicoproteína de oligodendrócito de mielina (IgG, ensaio baseado em células), e presença de evento desmielinizante (neurite óptica, mielite, síndrome do tronco cerebral, lesões corticais com convulsões). O espectro clínico desta doença desmielinizante inflamatória recém-reconhecida está se expandindo rapidamente. Faremos uma breve revisão das características epidemiológicas, manifestações clínicas, considerações diagnósticas e opções de tratamento da neurite óptica associada à glicoproteína de oligodendrócito de mielina-IgG.

## INTRODUCTION

Optic neuritis (ON) is one of the most important interfaces of ophthalmology and neurology. Ideally, neurologists and ophthalmologists should collaborate to document and interpret clinical manifestations, laboratory findings, and radiological features related to ON to increase the precision of diagnosis.

Since the Optic Neuritis Treatment Trial (ONTT)^(1)^ was published nearly 30 years ago, ON has been known to have a strong association with multiple sclerosis (MS), and corticosteroids have been shown to play a role in the acute management of ON. The ONTT showed the importance of magnetic resonance imaging (MRI) for estimating the risk of future development of MS and the effect of high-dose intravenous methylprednisolone (IVMP) for accelerating recovery of vision, although it has no effect on the long-term visual outcome.

In 2004^(2)^, after the anti-aquaporin-4 antibody (AQP4-IgG or NMO-IgG) was found in patients with severe ON and longitudinal extensive transverse myelitis (LEMT), neuromyelitis optica spectrum disease (NMOSD) was defined. AQP4-IgG is an important serological biomarker of ON^(3)^ that facilitates the differential diagnosis of NMOSD with MS. AQP4 is the most abundant water channel in the central nervous system (CNS), predominantly expressed at the end feet of astrocytes, thus making NMOSD a so-called astrocytopathy^(3)^.

Severe ON, which is frequently bilateral and recurrent and often has poor response to corticosteroids^(4,5)^, is the clinical hallmark of NMOSD. According to the latest diagnostic criteria for NMOSD^(6)^, NMOSD can be diagnosed even in the absence of AQP4-IgG in cases of extensive ON (>1/2 of the optic nerve length) or involvement of the optic chiasm, as observed on MRI, with normal brain MRI findings or the presence of only nonspecific white-matter lesions. Positivity for AQP4-IgG is highly specific (99%) to NMOSD. A relatively high sensitivity of 76% was attained when using a cell-based assay (CBA)^(7)^. Nevertheless, approximately one-third of patients who fulfill the NMOSD diagnostic criteria are AQP4-IgG negative^(8)^.

Approximately 20%-30% of patients with NMOSD who test negative for AQP4-IgG are seropositive for myelin oligodendrocyte glycoprotein antibodies (MOG-IgG)^(9,10)^. MOG-IgG reacts against a glycoprotein expressed on the myelin sheaths and oligodendrocyte processes (present exclusively in the CNS of mammals), probably with a structural function and possibly involved in the interaction between myelin and the immune system^(11)^. Even though MOG represents only 0.5% of the CNS myelin sheath, its epitopes seem to be highly immunogenic^(12)^. Both types of antibodies, anti-AQP4 and anti-MOG, eventually lead to the breakdown of the blood-brain barrier, CNS inflammation, and demyelination. However, MOG-IgG-associated disease (MOGAD) inflammation causes demyelination and primarily targets oligodendrocytes, whereas in NMOSD, severe astrocytic damage may lead to secondary demyelination and axonal loss. MOGAD has gained increasing attention, with a rapidly expanding clinical spectrum, and its existence seems to be associated with a specific demyelinating CNS disease that differs from MS and NMOSD.

The presence of recurrent and often bilateral ON is an important clinical hallmark of MOGAD, along with longitudinally extensive transverse myelitis (LEMT), which resembles NMOSD, thus complicating the differential diagnosis of chronic demyelinating diseases of the CNS. In fact, anti-MOG was found in 15% of a cohort with recurrent ON^(13)^. In addition, the association of anti--MOG-antibodies with acute demyelinated encephalomyelitis (ADEM), which are cortical lesions associated with epileptic seizures and brainstem symptoms, is well established. Furthermore, a clinical entity named chronic relapsing inflammatory ON (CRION), characterized by recurrent and steroid-dependent ON, shows a high association with MOG-IgG^(14)^.

In contrast to MS and NMOSD, which are conditions with well-established diagnostic criteria^(6,15)^, MOGAD still lacks definite diagnostic criteria owing to its recent discovery and expanding clinical spectrum. So far, two independent international panels have published recommendations on who should be tested for MOG-IgG and the timing of the testing^(16,17)^. Basically, these recommendations indicate that patients with bilateral and/or recurrent extensive ON and myelitis, or ON associated with optic disc swelling together with specific radiological and laboratorial characteristics should undergo prompt testing for anti-MOG, and if the result is positive, a diagnosis of MOGAD should be considered.

In the following sections, we summarize the current knowledge about MOGAD, emphasizing its ophthalmologic aspects. We will use the term *NMOSD* for anti-aquaporin 4-positive or anti-aquaporin 4- and MOG-IgG-negative cases that fulfill the latest NMOSD diagnostic criteria. We will use the term *MOGAD* for MOG-IgG-positive patients with demyelinating CNS events. This is of certain importance because in the international literature, MOGAD is often referred to as NMOSD.

### Epidemiology

MOGAD shows slight predominance among females, who account for approximately 63% of the cases^(18,19,20,21)^. However, this is less pronounced than in NMOSD, in which the female predominance is high (male-to-female ratio [M/F]=1:9^(22)^, and in MS (M/F=1:3). MOGAD has a wide range of ages at onset (1-81 years)^(18,19,21,23)^, with a mean age at onset of 31-37 years. This is slightly younger than mean age at onset in NMOSD (40 years^(24)^. Most cases occurred in Caucasians (56%-92%)^(19,21,25,26)^, which also contrasts with the cases of NMOSD, of which Asian and Afro-descendent individuals are highly represented^(22,24)^. However, the female predominance and racial differences in the incidence and prevalence of MOGAD are still controversial. Some investigators have advocated that the incidence of MOGAD has no sexual predilection and interracial differences^(27)^.

Up to 40% of NMOSD cases occur in association with other autoimmune disorders^(22)^, but in MOGAD, this association seems to be less frequently observed (11%)^(25)^. Data on populational incidence are scarce, but the incidence probably differs between the populations that have been studied. A recent study estimated that the nationwide MOG-IgG seropositivity rate in the Netherlands was 0.13 in 100,000 people per year for adults, with a higher incidence of MOG-IgG among children (0.31/100.000 people). An observational study conducted in Rio de Janeiro, Brazil, on a predominantly Afro-Brazilian (52%) cohort of NMOSD found MOG-IgG positivity in only 7% of AQP4-IgG-negative patients. The authors suggested the possibility of racial influence, with low positivity of MOG-IgG in Afro-descendants^(28)^.

## CLINICAL PRESENTATION

### Ophthalmologic evaluation

ON is the main clinical manifestation of MOGAD (present in 41%-63% of the cases), with high incidence rates of bilateral (24%-42%) and recurrent ON (64%)^(23)^. The ON recurrence rate in MOGAD is even higher than that in NMOSD (MOG vs NMOSD vs MS: annual relapse rate, 1.2% vs 0.6% vs 0.4%)^(13)^, and the second attack occurs more quickly after the initial attack in MOGAD (3.6 months) than in NMOSD (12.4 months) or MS (17 months)^(26)^.

Initially, ON in MOGAD may present with typical clinical ON characteristics such as moderate progressive loss of visual acuity (VA), visual field defects, dyschromatopsia, retro-orbital pain, and relative afferent pupillary defect. However, some anamnestic and clinical hints show that MOG-ON is an untypical form of ON, such as its bilateral manifestation and frequent relapses, as previously mentioned.

In MOGAD-ON, visual loss is often preceded by severe headache, which is sometimes reported as migraine-like^(29)^. Usually, patients who experience several ON episodes report specific headache characteristics associated with ON, and this information may be used to start pulse treatment early in ON, before visual loss occurs^(30)^. Pain during extraocular movements was reported in most patients (86%), a swollen optic disc is also common (86%), and bilateral simultaneous ON is present in 37%^(19)^. However, these are rarely found in MS-ON^(31)^. Approximately 20% of MOGAD relapses occur in a temporal association with recent vaccination or infectious disease^(25)^.

Some rare ophthalmologic manifestations of MOGAD, such as acute macular neuroretinopathy associated with acute ON, have been reported^(32)^. In addition, cases of ON associated with uveitis^(33)^, ON associated with macular star^(34)^, bilateral ON associated with bilateral serous detachment of the macula^(35)^, and ischemic optic neuropathy associated with diffuse orbital inflammation^(36)^ have also been reported. The clinical spectrum of MOGAD is likely to further expand over the coming years. Furthermore, the initial clinical phenotype seems to predict future relapses, as patients with onset of ON or myelitis have a greater likelihood of relapse in the same CNS area, and the risk of relapse has been found to increase with every new attack of ON^(37)^.

In addition to obtaining a detailed anamnesis, precise documentation of VA, visual field, pupillary reflex, and fundus changes, particularly on the optic nerve, during presentation and follow-up is fundamental for making a precise diagnosis and implementing the correct treatment for MOGAD.

#### VA and visual field

The range of VA at the onset of symptoms is wide (from 20/25 to no light perception). However, visual recovery is usually good (mean VA at follow-up, 20/30; ranging from 20/20 to NLP), and a poor visual outcome seems rare (with final VA of 20/200 or worse in 6% of cases)^(19)^. The visual prognosis in MOGAD seems to be better than that in NMOSD-ON^(13)^. This can be explained by the different pathological mechanisms of the two entities (“pure” demyelination in MOGAD vs. demyelination plus axonal injury in NMOSD). However, a residual visual function deficit often remains^(20,26)^. Owing to frequent relapses, long-term visual function impairment in MOGAD-ON can be comparable with that in NMOSD-ON^(38)^. No detailed reports have described the differences of specific visual field defects from other ON or specific visually evoked potential (VEP) findings in MOGAD, but the mean visual field defect seems to be worse in NMOSD-ON than in MOGAD-ON^(39)^. In MOGAD-ON, the visual field can show diverse patterns at baseline, including central and paracentral scotomas, temporal field cut, and diffuse visual field defects^(20)^. Altitudinal visual field effects, as observed in NMOSD-ON due to a possible vascular mechanism^(40)^, have not been reported in MOGAD-ON. VEP alterations (abnormal P100 latencies) were found in 60% of eyes with ON in patients with MOGAD in a small cohort, and all non-ON eyes presented a normal VEP^(38)^. However, in another study by the same group, P100 latency delay was observed in patients with MOGAD who presented with isolated LEMT but without any history of clinical manifestation of ON, which suggests the presence of subclinical optic nerve damage^(23)^. However, the “typical” VEP pattern in NMOSD, consisting of absent potential or reduced P100 amplitude with normal latency^(41)^, has not been reported in MOGAD so far. This may also be attributable to the different pathological mechanisms of the two entities.

### Optical coherence tomography

A recently published longitudinal optical coherence tomography (OCT) study in a cohort of MOG-positive patients in Germany^(42)^ showed no subclinical progressive thinning of the ganglion cell layer plus inner plexiform layer, in contrast to that observed in AQP4-seropositive NMOSD^(43)^ or MS cases^(44)^. However, pRNFL thinning was observed during follow-up in patients who tested positive for MOG-antibodies but who did not have any history of ON. A hypothesis of possible remission of a previous “subclinical” contralateral pRNFL edema was suggested, considering that an increase in pRNFL thickness was observed during clinical attacks, even in the non-involved eye^(42)^.

Another study showed better preservation of the pRNFL after ON in the eyes of patients with MOG-IgG antibodies than in those with AQP4-IgG antibodies. This was possibly due to the direct involvement of pathological antibodies in the inflammatory process in NMOSD-ON. However, nearly 80% of eyes with MOGAD-ON showed reduced RNFL on follow-up^(38)^. In approximately 20% of cases, the presence of macular microcysts in the inner nuclear layer led to increased macular volume in patients with MOGAD as compared with healthy subjects^(38)^ and is usually associated with optic nerve atrophy^(45)^. However, the prevalence of macular microcysts seems to have no significant difference between the eyes with MOGAD-ON and those with NMOSD-ON^(38)^. Thus, the presence of macular microcysts seems to be related to the severity of optic atrophy and not to a specific demyelinating CNS disease^(46)^. Furthermore, in the previous study, the authors concluded that the mean RNFL and visual field defect were better that VA as indicators of residual visual deficits after ON in MOGAD and NMOSD, as no significant difference in VA during the acute stage of ON was found between the MOG-IgG-and AQP4-IgG-positive patients^(39)^. A recent study found different OCT patterns of pRNFL loss in eyes with MOGAD, NMOSD, and MS-ON. The previously reported pRNFL thinning in the superior and inferior quadrants in NMOSD was not observed in the eyes with MOGAD-ON. In MOGAD-ON, mainly overall pRNFL thinning was observed, in contrast to the usually more pronounced temporal RNFL loss in MS. In addition, in eyes with MOGAD-ON, a discordance between the severity of inner retinal layer thinning and relative preserved visual outcome was observed and presumably was related to the different pathological mechanisms involved in these three demyelinating disorders^(47)^.

**Figure 1:**
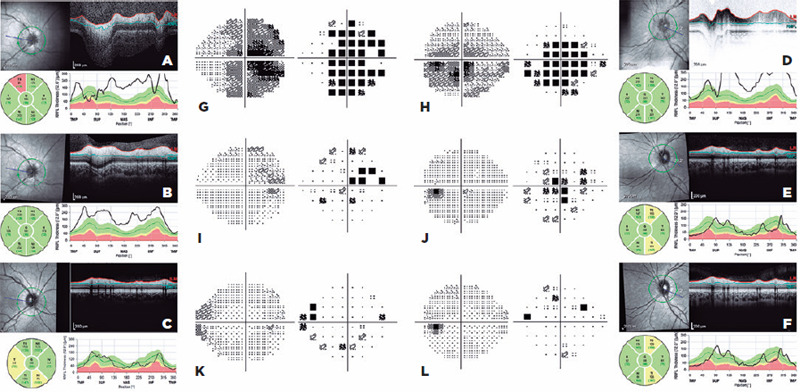
Visual field and OCT image of an 8-year-old girl who presented with multiphasic ADEM associated with LEMT and bilateral ON. A+D, G+H) presence of bilateral central scotoma and elevated optic disc, with a VA of a finger count of 30-cm distance. At this time, the patient was paraplegic and incontinent. IVMP was performed with partial clinical response. B+E, I+J) Ophthalmologic evaluation after 5 days of IVMP with partial regression of the central scotoma and papilledema. However, the patient was still severely handicapped, dependent on bilateral support for locomotion, and unable to read. PLEX was decided. C+F, K+L) Ophthalmologic evaluation 6 months after IVMP and PLEX, with complete regressions of central scotoma and papilledema, discrete predominantly temporal thinning of pRNFL, and visual acuity of 20/20. No neurological deficit and sphincter alterations were found on physical examination. The patient received rituximab therapy without relapses at 18-month follow-up.

ADEM: acute demyelinating encephalomyelitis; IVMP: intravenous pulse methylprednisolone; LEMT: longitudinal extensive transverse myelitis; ON: optic neuritis; OCT: optical coherence tomography; pRNFL: peripapillary retinal nerve fiber layer; PLEX: plasma exchange; VA: visual acuity.

Figure 1 shows an example of MOGAD-ON (visual field and OCT at the acute stage of clinical relapse and follow-up).

### Myelitis and encephalitis

In adults, myelitis is the second most prevalent manifestation (18%-47%) after ON in MOGAD. It is typically longitudinally extensive and similar to NMOSD. However, focal and MRI-negative myelitis have also been described in MOGAD^(48,49)^. Involvement of the conus medullaris, which presents with erectile and bladder dysfunctions, is more frequently observed in MOGAD than in NMOSD^(50)^ and MS.

In pediatric patients, an age-dependent bimodal predominant phenotype exists such that ADEM and ON are more frequently observed in younger children (aged 4-8 years) and older children (aged 10-13 years), respectively^(51)^. Furthermore, brainstem symptoms have been reported, consisting predominantly of area postrema syndrome with persistent nausea, vomiting, or hiccups in 15% of cases^(18,19,23,52)^. Until now, this has been considered a typical presentation of NMOSD. Involvement of cranial nerves (trigeminal, vestibulocochlear, or oculomotor)^(53)^ and relapsing lumbosacral myeloradiculitis^(54)^ have also been reported in MOGAD. Usually, patients experience several clinical relapses (most frequently consisting of ON), and the percentage of relapse-free patients diminishes with longer follow-up^(23,55,56)^. However, the prognosis in MOGAD is better than that in NMOSD, with good clinical recovery of both myelitis and ON^(57)^.

### Magnetic resonance imaging

MRI of the orbit and brain is certainly one of the most informative diagnostic tools when suspecting ON and helps in narrowing the differential diagnosis. Furthermore, it can provide additional prognostic information and guide treatment decisions. Bilateral optic nerve involvement has been well established to be more common in NMOSD and MOGAD than in MS, and chiasmal and optic tract involvements, especially if bilateral, are more associated with NMOSD^(58)^. MOGAD-ON affects predominantly the anterior parts of the optic nerve (retrobulbar and intraorbital), in contrast to NMOSD-ON, which more often shows intracranial optic nerve involvement^(19,49,58)^. MOGAD-ON rarely shows chiasmal involvement (12%)^(19)^.

Both MOGAD-ON and NMOSD-ON present with longitudinally extensive ON, usually compromising more than half of the optic nerve length^(19)^, with a median lesion length of 23.1 mm for both MOGAD-ON and NMOSD-ON, in contrast to the short ON lesions in MS (median length, 9.9 mm)^(58)^. Furthermore, perineural involvement (perineuritis) is frequently found in MOGAD (47%-50%)^(19,52)^. Brain MRI can also be used to differentiate MOGAD-ON from MS. A recent study showed a high prevalence (44%) of completely normal brain MRI (except optic nerve involvement) in MOGAD, and only 8% of MOG-IgG-positive patients fulfilled the 2010 McDonald diagnostic criteria for MS^(59)^.

### Cerebrospinal fluid

Cerebrospinal fluid (CSF) findings can also be used as a diagnostic tool to differentiate MOGAD-ON, principally MOGAD from MS. The typically encountered CSF alterations include discrete lymphocytic pleocytosis (26%-44%; approximately 25% of cases have >50 white blood cells), slightly elevated protein level (26%-42%), and absence of oligoclonal bands (OCB only present in 13% of MOGAD cases and 16% of NMOSD cases^(19,21,60,61)^, whereas in MS, OCB is present in most patients (>95%)^(62)^. Normal CSF findings are frequently observed in MOGAD-ON and, therefore, do not rule out a diagnosis of MOGAD^(61)^. Furthermore, the highly MS-specific MRZ reaction is absent in the CSF of patients with MOGAD^(61)^.

### Anti-MOG testing

Anti-MOG testing should be performed in the serum and not in the CSF because of its peripheral origin. Furthermore, only CBAs should be used. These can be used to recognize conformational MOG epitopes that are biologically relevant. CBAs are thus more specific than the previously used enzyme-linked immunosorbent assay and western blotting techniques. Experts have counseled that in cases of positive results, a second test should be performed using a different method, especially in slightly positive samples, to avoid false-positive results^(63)^. Double positivity for anti-AQP4 and anti-MOG antibodies seems to be extremely rare (1%). This usually presents a more aggressive course, resembling that of NMOSD^(64)^. As previously mentioned, since 2018, international recommendations for making diagnoses and performing antibody testing in MOGAD cases have existed. We recommend that this article should be read for further guidance^(16)^.

The implications of serial testing for anti-MOG are still controversial. The positivity for anti-MOG is known to fluctuate, and especially in pediatric cases of ADEM, MOG-IgG frequently becomes undetectable over 1-year follow-up. A recent study in Brazilian patients with MOGAD showed that the risk of relapse was associated with longitudinally persistent MOG-IgG seropositivity^(37)^.

Most patients who presented monophasic disease became spontaneously seronegative for MOG-IgG during long-term follow-up^(37)^. In a recent observational study in the UK, a quarter of the patients became MOG-IgG negative over time, and all the patients remained relapse-free^(18)^. Similar findings were reported by a pediatric MOGAD group, in which 24% of the patients who remained MOG-IgG positive after 6 months presented relapses, while none of the patients in the antibody-negative group relapsed^(65)^. The question of whom and when to retest for MOG-IgG, along with the therapeutic implications of this procedure, certainly requires study in greater depth and longitudinally, given that the relapse-free interval in MOGAD can extend over decades^(52)^. Table 1 summarizes typical clinical, epidemiological, and radiological findings in MOGAD, MS, and NMOSD.

**Table 1:** Comparison of demographic, clinical, and radiological differential features between MOGAD, NMOSD, and MS

@	MOGAD	NMOSD	MS
Neuropathology	Oligodendrocytopathy	Atrocytopathy	Demyelination, axonal injury, and astrogliosis
Disease course	Monophasic or relapsing (relapse-free up to decades), without disease progression between relapses	Relapsing, without disease progression between relapses	Relapsing, with secondary and primary progressions
Age at onset	Broad age of onset, children	Mean age at onset, 39 years	20–30 years
Sex	Slight female predominance	Female-to-male ratio = 9:1	Female-to-male ratio = 3:1
Ethnicity	Predominantly Caucasian	Overrepresented in Asian and Afro-Caribbean races	Predominantly Caucasian
ON involvement	BilateralExtensive (median length 23,1 mm)	Bilateral Extensive (median length, 23.1 mm)	Unilateral Short segment (median length, 9.9 mm)
ON localization	Anterior predominance Papilitis (86%) Perineural involvement	Posterior predominance with chiasma and optic tract involvement	Predominantly retrobulbar ON
Ocular pain during ON attack	86%	19%	44%
Final visual outcome	Initially good, worsens with recurrence of ON	Usually important visual sequela	Good
Coexisting autoimmune disorder	Rare	Frequent	Rare
CSF findings	OCBs are rare (around 10%) Pleocytosis is common Lactate and protein levels are elevated	OCBs are rare (around 10%), pleocytosis is common, and lactate and protein levels are elevated	OCBs are common (>95%) Moderate pleocytosis Normal lactate and protein levels
Brain MRI findings	Can have normal brain MRI findings; ADEM; poorly demarcated lesions (“fluffy lesions”); pons; cerebellar peduncles; and cortical lesions	No brain lesions typical of MS; brainstem/pons/diencephalic lesions	Multiple focal white-matter lesions, ovoid lesions adjacent to body of the lateral ventricles, Dawson finger, and T1 hypointense lesions
Spinal MRI findings	Usually long segment lesions (>3 vertebral segments); short lesions in up to 25%; involvement of conus	Long segment lesions (>3 vertebral segments); dorsal brainstem lesions continuous with cervical cord lesions	Short lesions, peripheral localization, and predominantly cervical medulla
Treatment	Immunosuppressive	Immunosuppressive	Immunomodulatory and immunosuppressive

ADEM= acute demyelinating encephalomyelitis; CSF= cerebrospinal fluid; MOGAD= MOG antibody disease; MS= multiple sclerosis; NMOSD= neuromyelitis optica spectrum disorders; OCB= oligoclonal bands; ON= optic neuritis.

## TREATMENT

So far, no randomized clinical trials have been conducted to guide treatment of MOGAD cases. Therapeutic decisions are basically extrapolated from data on other CNS autoimmune diseases, especially NMOSD, as the disease-modifying drugs used in MS are not effective in MOGAD^(66,67)^. The therapeutic strategies for MOGAD can be divided into treatment of acute relapses and prevention of relapses in the form of continuous prophylactic treatment. As mentioned earlier, the role of corticosteroids in acute ON is mainly based on the beneficial effect of steroids on the visual recovery observed in the ONTT. However, the main study population in the ONTT consisted of MS-ON cases, and only 1.7% (3/177) of the participants in the ONTT tested positive for MOG-IgG, and none tested positive for anti-AQP4^(68)^.

The treatment strategy usually implemented consists of intravenous methylprednisolone (IVMP) pulses (e.g., 1000 mg of MP over 3-5 days) as soon as possible after MOGAD-ON has been diagnosed. A recent article examined the detrimental effect of postponing intravenous steroid treatment in cases of acute ON in NMOSD and MOGAD. As previously shown, retinal ganglion cell layer loss starts within a few days after ON and possibly predicts future visual loss^(69)^. Administration of IVMP treatment on day 4 or earlier was identified as the cutoff point for regaining 20/20 vision (odds ratio for failure, 8.33), and withholding treatment for >7 days had an odds ratio of 10.0 for failure to recover 20/30 vision, for both NMOSD-ON and MOG-ON. These findings highlight the importance of implementing, if feasible, a so-called hyperacute IVMP treatment (treatment within 2 days of symptom onset) and inhibiting the intensive inflammatory process that precedes axonal degeneration^(70)^. Several reports have shown that MOGAD-ON responds well to IVMP (almost complete recovery in 50%)^(23)^. However, if no significant recovery of visual function is observed, therapeutic plasma exchange (PLEX; usually a total of 5 sessions on alternating days) should be performed without delay.

In observational studies with relapses of NMOSD, including ON, use of PLEX (with or without preceding IVMP) seems to be more effective than IVMP alone, and apparently PLEX is more effective the sooner it is started^(5)^. A potential beneficial effect was found even in cases of delayed PLEX (interval between symptom onset and PLEX of >30 days)^(71)^. If visual function does not respond to IVMP satisfactorily, PLEX could be considered even as late as 6 weeks after symptom onset in cases of severe and steroid-resistant ON^(72)^.

Concerning long-term immunosuppressive treatment, no randomized studies have been conducted on MOGAD. Oral steroids seem to be useful for preventing relapses^(59)^, at least partially. However, because of their harmful long-term effects, transition to other immunosuppressive drugs such as azathioprine (often used as first-line therapy), mycophenolate mofetil, and rituximab is necessary. These therapies are recommended in the international NMOSD guidelines, and these medications also seem to have a favorable impact on the clinical outcomes of MOGAD cases^(55)^. Rituximab is not as effective for preventing relapses in MOGAD as in NMOSD and MS, despite robust B-cell depletion^(73,74)^.

In an observational study with 102 children who presented with relapsing demyelinating syndrome such as NMO-SD, ADEM, or relapsing ON who tested positive for MOG-IgG, the effectiveness of monthly treatment with IVIG was found to be superior to that of other treatments^(67)^. Thus, the treatment option is more attractive for avoiding immunosuppression in this age group. Single-case reports on adults who were successfully treated with IVIG underline this therapeutic option^(75,76)^, especially in refractive cases.

MOGAD, which, perhaps, should be more accurately named “MOGAD spectrum disorder”, is a rapidly expanding CNS demyelinating disorder with different clinical manifestations. It is predominantly characterized by relapsing ON (often simultaneously bilateral), myelitis, ADEM, and brainstem symptoms resembling MS and NMOSD. For patients who present to ophthalmologists with relapsing and/or bilateral ON that responds well to steroids or with ON associated with elevated optic disc, a preceding headache, and a recent history of infection or vaccination, serological testing for MOG-IgG should be considered.
